# Stage-dependent choriocapillaris impairment in Best vitelliform macular dystrophy characterized by optical coherence tomography angiography

**DOI:** 10.1038/s41598-021-93316-3

**Published:** 2021-07-12

**Authors:** Ruben Jauregui, Rait Parmann, Yan Nuzbrokh, Stephen H. Tsang, Janet R. Sparrow

**Affiliations:** 1 Department of Ophthalmology, Edward S. Harkness Eye Institute, Columbia University Irving Medical Center, 635 West 165th Street, Box 212, New York, NY 10032 USA; 2 Jonas Children’s Vision Care, New York, NY USA; 3 Department of Pathology and Cell Biology, Columbia University Irving Medical Center, New York, NY USA

**Keywords:** Medical research, Genetics research, Outcomes research

## Abstract

Characterization of vascular impairment in Best vitelliform macular dystrophy (BVMD) is essential for the development of treatment modalities and therapy trials. As such, we seek to characterize the choriocapillaris (CC) at each stage of the disease process in 22 patients (44 eyes) with a diagnosis of BVMD confirmed by genetic sequencing. We utilize optical coherence tomography angiography (OCTA) images to characterize the CC and correlate our findings to the status of the retinal pigment epithelium (RPE) as observed on short-wavelength fundus autofluorescence (SW-AF) images. We observed that in the vitelliruptive stage, the CC appeared as bright and granular in the area where the vitelliform lesion was present. In the atrophic stage, varying degrees of CC atrophy were observed within the lesion area, with the regions of CC atrophy appearing as hypoautofluorescent on SW-AF images. Our results suggest that the CC impairment observed in the vitelliruptive stage of BVMD progressively culminates in the CC atrophy observed at the atrophic stage. As such, OCTA imaging can be used to characterize CC impairment in BVMD patients as part of diagnosis and tracking of disease progression. Our findings suggest that the best window of opportunity for therapeutic approaches is before the atrophic stage, as it is during this stage that CC atrophy is observed.

## Introduction

Best vitelliform macular dystrophy (BVMD, OMIM: 607854) is most commonly inherited in an autosomal dominant manner and caused by mutations in the gene *BEST1* (11q13), with over 200 mutations reported up to date^1–3^. The gene *BEST1* encodes the integral membrane protein bestrophin-1, a chloride channel that is located in the basolateral membrane of retinal pigment epithelium (RPE) cells and is activated by cytosolic calcium^4–8^. The typical clinical presentation of BVMD involves the development of a yellow vitelliform or egg-yolk-like macular lesion at a young age that regresses with time leading to atrophic changes in the RPE and loss of central visual acuity^1,9^. Classically, the disease process is divided into five stages: previtelliform, vitelliform, pseudohypopyon, vitelliruptive, and atrophic^1^. Initially, no lesions are observed on the posterior pole and vision remains normal (previtelliform). This stage is followed by the development of the vitelliform lesion, which might cause a mild decrease in vision (vitelliform), and then followed by the partial fluid resorption of the lesion (pseudohypopyon)^1^. The vitelliform lesion eventually “scrambles” (vitelliruptive stage), potentially causing a substantial decrease in vision^1^. The final stage involves atrophic changes to the RPE. The clinical diagnosis of BVMD is made by a combination of fundoscopy of the posterior pole, detection of a pathogenic variant in *BEST1*, and an abnormal light-rise on electrooculography (EOG).

Optical coherence tomography angiography (OCTA) is a non-invasive imaging modality that provides images of the retinal and choroidal vasculature^10^. Multiple studies have used OCTA to characterize the retinal and choroidal blood flow in retinal dystrophies such as retinitis pigmentosa, recessive Stargardt disease, and choroideremia^11–15^. For BVMD in particular, OCTA studies have shown retinal vascular impairment in patients and have analyzed its use in detecting choroidal neovascularization (CNV), a potentially severe complication of BVMD estimated to occur in 2–9% of cases^9,16^.

In this study, we explore the use of OCTA imaging in assessing the degree of choriocapillaris (CC) impairment in patients with BVMD and relate these changes to the status of the RPE as demonstrated on short-wavelength fundus autofluorescence (SW-AF) images.

## Methods

### Patients

The study procedures were defined as outlined by the protocol #AAAB6560 approved by the Institutional Review Board at Columbia University Irving Medical Center. Given the retrospective nature of the study and minimal risk conferred to patients, the need for informed consent was waived. The study adhered to the tenets of the Declaration of Helsinki. The data presented in this study, including images and genetic sequencing results, are not identifiable to individual patients.

We conducted a retrospective review of patients with a diagnosis of BVMD at the Harkness Eye Institute. The diagnosis was made by S.H.T. based on a combination of clinical presentation, genetic sequencing, and EOG testing. Inclusion criteria for this study included (1) the diagnosis of BVMD confirmed by genetic sequencing and (2) a clinical visit where spectral-domain optical coherence tomography (SD-OCT), short-wavelength fundus autofluorescence (SW-AF), and OCTA images where obtained.

### Image acquisition and analyses

Clinical examination and imaging across all modalities were conducted after pupillary dilation (> 7 mm) with phenylephrine hydrochloride (2.5%) and tropicamide (1%). SD-OCT and SW-AF (488 nm excitation) images were acquired with the Spectralis HRA + OCT (Heidelberg Engineering, Heidelberg, Germany), while OCTA images with co-registered SD-OCT images were acquired using the Zeiss AngioPlex Cirrus HD-OCT 5000 (Zeiss Meditec Inc, Dublin, California, USA). Images of the CC via OCTA were obtained by automated segmentation of full-thickness retina scans into different vascular layers, performed by the OCTA software. After layer segmentation, the software identifies the SD-OCT band that corresponds to the RPE and defines the CC slab as having an inner surface 29 μm below the RPE and an outer surface 49 μm below the RPE, creating a uniform thickness of 20 μm. The automatic layer segmentation for each image was reviewed to ensure accuracy by both authors R.J. and R.P. Adjustments were made to 6 images.

Both eyes from each patient were analyzed. All images from the three modalities were analyzed independently by two graders (R.J. and R.P.). The eyes were staged according to SD-OCT and SW-AF findings as previously published^17^. Inter-grader agreement was quantified by using Fleiss’ kappa, a statistical measure to assess agreement between graders classifying items. Our calculated κ was 0.87 (95% confidence interval: 0.67–1.00), indicating a high-level of agreement. For the eyes where the graders disagreed regarding staging, both graders discussed the cases and reached a consensus.

## Results

A total of 22 patients (44 eyes) were analyzed for this study. The average age for the cohort was 47 ± 17 years (mean ± standard deviation). One eye presented at the previtelliform stage, 3 eyes presented at the vitelliform stage, 7 eyes presented at the pseudohypopyon stage, 18 eyes presented at the vitelliruptive stage, and 15 eyes presented at the atrophic stage. A total of 4 patients presented with stage discordance between fellow eyes. None of the patients presented with or had a history of CNV. Demographical, clinical, and genetic sequencing information is detailed in Table 1.

**Table 1:** Demographical, clinical, and genetic sequencing information of the patient cohort analyzed in the study.

Patient ID	Age	Disease stage		BCVA		*BEST1* variant	
		OD	OS	OD	OS	cDNA	Protein
1	33	Pseudohypopyon	Pseudohypopyon	20/70	20/70	c.473G > A	p.R158H
2	53	Vitelliruptive	Vitelliruptive	20/80	20/70	c.253T > C	p.Y85H
3	57	Atrophic	Atrophic	20/100	20/60	c.253T > C	p.Y85H
4	43	Vitelliruptive	Vitelliruptive	20/50	20/50	c.877C > A	p.Q293K
5	73	Pseudohypopyon	Atrophic	20/60	20/400	c.896G > A	p.Q299E
6	23	Vitelliruptive	Vitelliruptive	20/20	20/20	c.887A > G	p.N296S
7	61	Atrophic	Atrophic	20/100	20/100	c.887A > G	p.N296S
8	57	Atrophic	Atrophic	20/70	20/70	c.89A > G	p.K30R
9	49	Vitelliruptive	Atrophic	20/50	20/60	c.900G > C	p.E300D
10	60	Vitelliruptive	Vitelliruptive	20/40	20/50	c.274C > T	p.R92C
11	32	Vitelliruptive	Vitelliruptive	20/50	20/30	c.727G > A	p.A243T
12	77	Atrophic	Atrophic	20/80	20/80	c.602T > C	p.I201T
13	43	Vitelliruptive	Vitelliruptive	20/30	20/20	c.652C > T	p.R218C
14	59	Vitelliruptive	Vitelliruptive	20/150	20/70	c.653G > A	p.R218H
15	45	Vitelliform	Vitelliform	20/30	20/25	c.653G > A	p.R218H
16	12	Pseudohypopyon	Pseudohypopyon	20/80	20/25	c.28G > A	p.A10T
17	44	Vitelliform	Previtelliform	20/25	20/20	c.884T > C	p.I295T
18	42	Atrophic	Atrophic	20/400	20/400	c.218T > A	p.I73N
19	34	Vitelliruptive	Vitelliruptive	20/20	20/20	c.727G > A	p.A243T
20	60	Atrophic	Atrophic	20/125	20/125	c.250T > G	p.F84V
21	15	Pseudohypopyon	Pseudohypopyon	20/20	20/20	c.701T > C	p.L234P
22	57	Atrophic	Vitelliruptive	20/400	20/80	c.652C > T	p.R218C

*BCVA* Best-corrected visual acuity.

**Figure 1:**
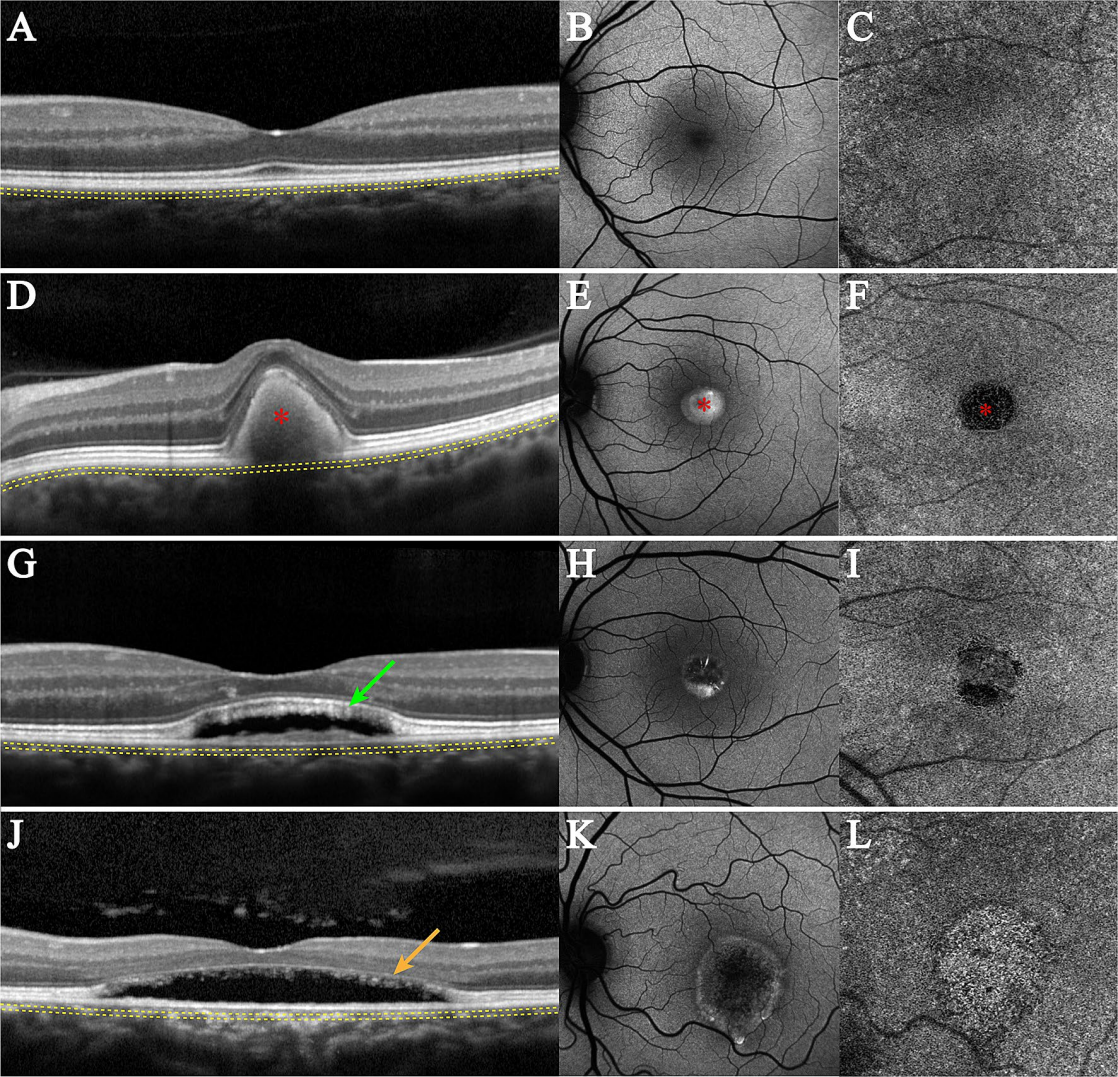
Multimodal imaging of eyes at the previtelliform, vitelliform, pseudohypopyon, and vitelliruptive stages. At the previtelliform stage, spectral-domain optical coherence tomography (SD-OCT) images revealed intact retinal layers (**A**). Short-wavelength fundus autofluorescence (SW-AF) images demonstrated macular hypoautofluorescence (hypoAF) while images of the choriocapillaris (CC) by optical coherence tomography angiography (OCTA) demonstrated a homogenous pattern, both as observed in healthy eyes (**B**,**C**). At the vitelliform stage, a hyperreflective material (red asterisk) is observed in SD-OCT images (**D**). This material is hyperautofluorescent (hyperAF) in SW-AF images (**E**) and obstructs the OCTA signal, causing the area to appear as dark and devoid of CC (**F**). The eyes at the pseudohypopyon stage presented with hyporeflective subretinal fluid and a thickened interdigitation zone (IZ) band with abnormal reflectance (green arrow) in SD-OCT images (**G**). On SW-AF images, patchy hypoAF was observed in the macula, with inferior displacement of the hyperAF material (**H**). The CC corresponding to the lesion area appeared heterogeneous, with regions where the CC was visible and regions where it was not (**I**). Eyes at the vitelliruptive stage also presented with hyporeflective fluid on SD-OCT images, but the IZ band appeared as fragmented, with the remaining outer segments appearing in clumps (orange arrow) (**J**). SW-AF images revealed macular hypoAF, whereas the CC appeared granular and bright on OCTA images (**K**,**L**). The yellow dashed lines on the SD-OCT images represent the approximate location of the CC slab.

In the eye at the previtelliform stage, SD-OCT imaging revealed intact retinal layers with no abnormalities, while SW-AF imaging demonstrated macular hypoautofluorescence (hypoAF) as observed in healthy eyes (Fig. 1A,B). Similarly, the CC appeared homogenous, as observed in healthy subjects (Fig. 1C).

In the eyes at the vitelliform stage, a hyperreflective, macular vitelliform material was observed in SD-OCT imaging, which appeared as hyperautofluorescent (hyperAF) on SW-AF images (Fig. 1D,E). OCTA images revealed a dark area with no CC visualized; this anomaly corresponded to the area of the vitelliform lesion (Fig. 1F).

The eyes at the pseudohypopyon stage presented with significant hyporeflective subretinal fluid with variable degrees of accumulated hyperreflective material on SD-OCT. The interdigitation zone (IZ) line, corresponding to photoreceptors’ outer segments, was thickened with abnormal reflectiveness (Fig. 1G). In SW-AF images, inferior displacement of the hyperAF material was observed (Fig. 1H). In OCTA images, the area of the CC corresponding to the lesion appeared heterogeneous, with the CC non-visible in some parts of the lesion and visualized in others (Fig. 1I).

The 18 eyes at the vitelliruptive stage similarly presented with subretinal fluid and hyperreflective material in SD-OCT scans. The IZ line was shortened and fragmented, with the remaining outer segments misaligned and clumped in groups (Fig. 1J). In SW-AF images, the lesion appeared as hypoAF centrally, with varying amounts of the hyperAF vitelliform material among different patients (Fig. 1K). On OCTA images, the area of the CC corresponding to lesion appeared as bright and with increased granularity (Fig. 1L).

**Figure 2:**
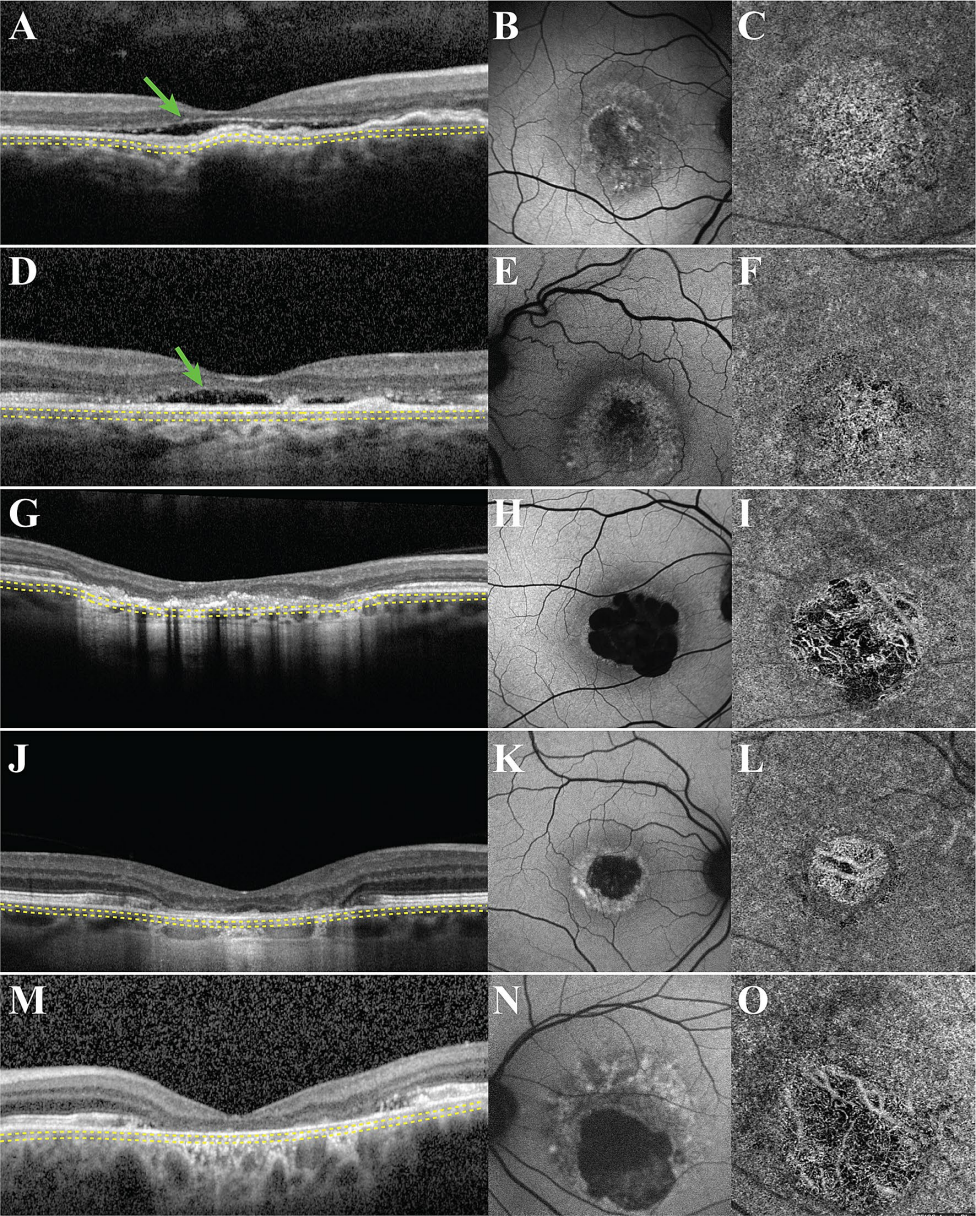
Multimodal imaging of eyes at the atrophic stage. Spectral-domain optical coherence tomography (SD-OCT) images revealed two different presentations of the atrophic stage. One presentation involves shallow, hyporeflective fluid, with loss of the interdigitation zone (IZ) band (green arrow) (**A**,**D**). The other presentation involves loss of outer retinal layers with subsequent collapse of the inner retinal layers (**G**,**J**,**M**). All of these lesions in SD-OCT images presented with increased signal transmission into the choroid. In short-wavelength fundus autofluorescence (SW-AF) images (**B**,**E**,**H**,**K**,**N**), these lesions corresponded to hypoautofluorescent (hypoAF) areas, with denser hypoAF observed in patients with collapse of the inner retinal layers on SD-OCT images (**H**,**K**,**N**). In OCTA images, the aforementioned lesions on SD-OCT and SW-AF imaging appeared as areas of bright choriocapillaris (CC), with varying degrees of atrophy. In the eyes with hyporeflective fluid, CC atrophy was patchy (**C**,**F**), whereas in the patients with collapse of the inner retinal layers, CC atrophy was more severe, with the underlying choroidal vessels visible (**I**,**L**,**O**). The yellow dashed lines on the SD-OCT images represent the approximate location of the CC slab.

The 15 eyes at the atrophic stage presented with loss of the outer retinal layers on SD-OCT imaging (Fig. 2). Six of these eyes presented with shallow, hyporeflective subretinal fluid, with disappearance of the majority of the IZ line (Fig. 2A,D). The remaining 9 eyes presented with complete resorption of the subretinal fluid, disappearance of the outer retinal layers, and subsequent collapse of the inner retinal layers (Fig. 2G,J,M). The areas corresponding to the lesion in all 15 eyes demonstrated increased signal transmission into the choroid in SD-OCT images, suggestive of RPE atrophy. Furthermore, the lesion areas appeared as hypoAF on SW-AF imaging, further indicating RPE damage (Fig. 2B,E,H,K,N). All 15 eyes included in this cohort presented with varying degrees of CC atrophy corresponding to the hypoAF areas observed in SW-AF images (Fig. 2C,F,I,L,O). In eyes where there was incomplete resorption of the subretinal fluid, patchy CC atrophy was observed, whereas in the eyes with complete fluid resorption and subsequent collapse of the outer retinal layers, more severe CC atrophy was present to the extent that the underlying larger choroidal vessels were visible.

## Discussion

The non-invasive nature of OCTA has allowed clinicians and researchers to study and characterize changes in the retinal and choroidal vasculatures in a variety of disease processes. A previous study by Battaglia Parodi and colleagues reported significant retinal vascular impairment in eyes of BVMD patients^16^. In particular, they noted a decrease in vessel density in both the superficial (SCP) and deep capillary plexuses (DCP) in patients with BVMD disease in the pseudohypopyon, vitelliruptive, and atrophic stages, as compared to controls^16^. Significant enlargement of the foveal avascular zone in both SCP and DCP was also reported in patients at the atrophic stage^16^. Similarly, Guduru and colleagues also reported an abnormal foveal avascular zone and patchy vascularity loss in both SCP and DCP layers^9^. Furthermore, they analyzed the use of OCTA in detecting CNV in BVMD patients and classified it into four different patterns^9^. Various studies have demonstrated the essential role of RPE-derived vascular endothelial growth factor (VEGF) in the development and maintenance of the CC^18–20^. As such, we were interested in characterizing changes in the CC of BVMD patients as observed in OCTA images and to correlate these changes with the status of the RPE as noted in SW-AF images.

Based on our study cohort, we observed that the phenotype of the CC correlates with the stage of the disease process. In the previtelliform stage, the CC was comparable to those observed in healthy subjects. In the vitelliform and pseudohypopyon stages, there was a masking effect on the CC due to the vitelliform lesion, and thus we observed a dark area on OCTA imaging. Furthermore, in the pseudohypopyon eyes, the lesion area contained regions where the CC was visible and regions where it was not. We speculate that this effect is due to the heterogenous mixture of vitelliform material and fluid, as in this stage there is resorption of liquid from the vitelliform material. Given the masking effect created by the lesion at the vitelliform and pseudohypopyon stages, OCTA cannot fully characterize the state of the CC in these two stages.

As the material is resorbed throughout the vitelliruptive and atrophic stages, we were able to discern stage-specific CC phenotypes. In the vitelliruptive stage, we observed an area of brighter and granular CC. The vasculature in OCTA images is constructed based on the flow of red blood cells, with brighter areas signifying increased blood flow^10^. We suggest that this observed brightness in our study is not due to increased blood flow, but rather an indirect effect of the disappearance of the overlying RPE. As the overlying RPE atrophies, the area receives more signal from the OCTA modality as compared to other regions, and thus the area of CC appears qualitatively brighter. A brighter CC was observed in both the vitelliruptive and atrophic stages. This change in the CC appearance occurred in parallel with the progressive atrophy of the RPE as suggested by the progressive hypoAF of the lesion area in SW-AF images. Although no obvious CC atrophy is observed in the vitelliruptive stage, the increased CC granularity in the lesion area suggests the beginning of morphologic changes and possible impairment. In the atrophic stage, the lesion area appeared as densely hypoAF in SW-AF images, indicating RPE atrophy. In the eyes where hyporeflective fluid was visible in SD-OCT scans, the lesion area was not as densely hypoAF as compared to the eyes where the outer retinal layers have atrophied and inner retinal layers subsequently collapsed. This presentation parallels the findings in OCTA images. In those patients with remaining hyporeflective fluid, CC atrophy was observed as patchy, but in the patients with collapse of the inner retinal layers and dense hypoAF, CC atrophy was more severe to the extent that the underlying choroidal vasculature was visible. Despite being classified as eyes in the atrophic stage, we suggest that those eyes with remaining hyporeflective fluid reflect an intermediate stage between the vitelliruptive and atrophic stages, as suggested by the findings in SD-OCT, SW-AF, and OCTA images. Given the important relationship between the RPE and CC, we believe that as the disease progresses and the RPE atrophies, there is subsequent atrophy of the CC. We observed that CC changes begin on the vitelliruptive stage and progress throughout the atrophic stage, culminating in atrophy. An important limitation of the study, however, is that we do not report prospective data, throughout which we could observe the progression of individual patients through the different stages. Future studies should look to characterize the progression of BVMD with OCTA imaging.

With the goal of increasing the feasibility of applying our results in a clinical setting by those attending to BVMD patients, we chose to use the automatic segmentation from our OCTA software for defining the CC slab. The software defines the CC slab as having an inner surface 29 μm below the RPE and an outer surface 49 μm below the RPE, creating a uniform slab thickness of 20 μm. Defining a CC slab that is true to the anatomy and accurately depicts the CC is important, but given the technical limitations of current OCTA technology and the relative thinness of the retinal/choroidal layers, there is no uniform consensus for CC segmentation guidelines in the literature. The guidelines outlined by Chu et al*.* for imaging and quantifying different CC parameters suggest that a slab of 10–20 μm in thickness yields the best OCTA *en face* images, similar to our CC slab^21^. In a different study, Byon et al*.* reported that the most consistent measurements of CC flow deficits are obtained from images generated by using a 21–31 μm slab below the RPE segmentation line^22^. Furthermore, the authors elaborate that 21–31 μm slab is better referred to as the “inner choroidal” slab, as the CC image from this slab may also contain other choroidal structures^22^. Given that our choice of CC slab is deeper and thicker than the 21–31 μm slab used in their study, the CC images presented in this study also likely contain deeper choroidal structures in addition to the CC, which is a weakness of our study and others that have used either the 29–49 μm or deeper slabs^23–28^. Nevertheless, more studies are needed to establish and validate uniform metrics for defining the CC slab.

Our results can be applied by clinicians, researchers, and those participating in the development of potential future treatments. For clinicians and researchers, the phenotypes observed on OCTA images and their correlation to SD-OCT and SW-AF images can help in the diagnosis of and stage determination in BVMD patients. We observed CC changes beginning at the vitelliruptive stage that culminate in CC atrophy at the atrophic stage. As such, these findings have important implications for the development of future treatment modalities for BVMD. Given the autosomal dominant nature of BVMD, gene supplementation therapy, effectively demonstrated for *RPE65*-Leber congenital amaurosis, will likely not be successful as gene replacement will not counteract the effect of gain-of-function mutants in BVMD^29–32^. Yet, other potential treatment modalities have been explored by various groups. For example, autologous transplantation of induced pluripotent stem cells (iPSC)-derived RPE is a promising therapy. Furthermore, a clinical trial in Japan is using iPSC-RPE to treat exudative age-related macular degeneration^33^. In regards to BVMD, RPE derived from autologous iPSC would carry the same pathogenic variant in *BEST1*, and thus CRISPR/Cas9 gene editing technologies would be necessary to correct this variant in the iPSC-RPE cells^34^. Despite these potential treatment modalities, our results demonstrate that the atrophic stage of BVMD presents with atrophied CC, which would pose an additional challenge for a treatment modality, as healthy CC are needed to support the RPE cells. These findings would suggest that potential treatment modalities would be most effective before patients reach the atrophic stage.

## Data Availability

The datasets generated during and/or analyzed during the current study are available from the corresponding author on reasonable request.
